# The application of the Chinese version of the Body Image Disturbance Questionnaire in patients with systemic lupus erythematosus

**DOI:** 10.1097/MD.0000000000024849

**Published:** 2021-02-19

**Authors:** Haoyang Chen, Xin Chen, Hongyan Yan, Jielin Ben, Xiaoyan Yao, Pingyu Yang, Minhua Zhang, Tiantian Jin, Biyu Shen

**Affiliations:** a Department of Nursing, Affiliated Hospital 2 of Nantong University; b Medical College (Nursing School) of Nantong University, Nantong, China.

**Keywords:** Body Image Disturbance Questionnaire, reliability, systemic lupus erythematosus, validity

## Abstract

This study aimed to translate the Body Image Disturbance Questionnaire (BIDQ) into Chinese and evaluate its reliability and validity in a sample of patients with systemic lupus erythematosus (SLE).

Following the translation and revision of the Chinese version of the BIDQ, 169 patients with SLE were chosen as respondents to test the questionnaire's reliability and validity. We tested the content's validity through expert group evaluation. It is structural validity was examined through exploratory factor analysis and confirmatory factor analysis, and reliability was evaluated using Cronbach's α and test-retest reliability.

The Chinese version of the BIDQ showed a content validity of .92. A two-factor structure was revealed by exploratory factor analysis, which explained 67.83% of the variance and proved by confirmatory factor analysis. Its overall Cronbach's α was .82 *(P* *<* .001), and the Cronbach's α for each item ranged from .76 to .83. The test-retest reliability was .82, with the Cronbach's α for each item ranging from .76 to .84.

Thus, adequate reliability and validity of the Chinese version of the BIDQ were demonstrated for use in patients with SLE.

## 1 Introduction

Body image is a term used to describe individuals’ perceptions of their appearance, body functions, and body state. The construct comprises 2 components—the objective evaluation of one's own body image and one's subjective feelings regarding this body image.^[1]^ In situations in which one of the 2 aspects is damaged, a body image disturbance (BID) can occur.^[2]^

Systemic lupus erythematosus (SLE) is an autoimmune disease that invades multiple organs and systems that is characterized by symptoms including changes in gait and/or posture, rashes, loss of skin pigmentation, scarring, skin damage, and butterfly-like erythema.^[3]^ SLE is common among young and middle-aged women compared to other age groups. The treatments for SLE can result in visible side effects. For older female patients playing important roles in family, work, and society, these changes may contribute to shifts in their body image, which, in turn, could increase the pressure they experience in their daily lives. Studies have found that patients diagnosed with SLE who also have BID report having high levels of anxiety and depression, which can affect their quality of life.^[4]^

Much of the previous research on BID has been conducted on patients with eating disorders or cancer.^[5]^ Research on cancer patients has indicated that poor body image negatively influences their quality of life.^[6]^

The Body Image Disturbance Questionnaire (BIDQ) is the most commonly used questionnaire for evaluating body image^[7,8]^ and has been found to have good reliability and validity with samples of people with eating disorders,^[9]^ orthognathic surgery,^[10]^ and obesity.^[11]^ However, the reliability and validity of the Chinese version of the BIDQ have only been established with a sample of patients with ankylosing spondylitis in China.^[12]^ Its use has not been validated in SLE patients. The present study aimed primarily to evaluate the reliability and validity of the Chinese version of the BIDQ with a sample of patients with SLE. As there is public concern regarding the well-being of patients with SLE, it would be beneficial to understand the presence of BID in this population, which would allow clinicians to provide timely guidance and care to reduce the psychological burden of patients as well as their families and communities.

## 2 Methods

### 2.1 Participants

Patients with a diagnosis of SLE were recruited from Nantong, China, between March 2018 and September 2019. A total of 169 patients with SLE were invited to participate in the study. All participants met the 1997 American College of Rheumatology revised criteria for the classification of SLE.^[13]^ Patients were excluded from participation if:

1. they failed to complete the questionnaire, or
2. they had a comorbid diagnosis (e.g., serious infections or cardiac, respiratory, gastrointestinal, neurological, or endocrine disease) that could influence SLE activity.

The sample size was estimated based on the rule that the number of participants should be at least 5 times greater than the total number of items on the scale.^[14]^ Furthermore, according to statistical factor analysis requirements, exploratory factor analysis (EFA) and confirmatory factor analysis (CFA) used different samples.^[15]^ Seven items are on the BIDQ. A total of 161 valid questionnaires were recovered; of these, 54 participants (Sample 1) were selected for the EFA, and the remaining 117 participants (Sample 2) were used for the CFA.

### 2.2 Body Image Disturbance Questionnaire

Body image was assessed using the BIDQ containing 7 scaled items, with each scored from 0 (not affected) to 8 (extremely affected). Items included appearance-related concerns (BIDQ1); mental preoccupation (BIDQ2); emotional distress (BIDQ3); social, occupational, or functional impairment (BIDQ4); social life interference (BIDQ5); educational, occupational, or other functional interferences (BIDQ6); and behavioral avoidance (BIDQ7).

### 2.3 The translation process

We administered the BIDQ after receiving consent from the respondents. We established a translation team, and 2 bilingual translators with master's degrees (1 with a medical background and the other without) created 2 independent simplified Chinese translations. After the BIDQ was translated, all versions were consolidated into a single translation. Two independent bilingual translators reviewed the consolidated translation—both were native Chinese speakers, 1 having a doctorate in nursing in Thailand, and the other being a professional English translator.

### 2.4 Harmonization and proofreading

After a comparative analysis with the original scale, the differences in the translated version were discussed, and corresponding revisions were made before finalizing it to ensure the accuracy of the scale translation.

### 2.5 Expert review to finalize phrasing for linguistic validation

Due to the differences between clinical environments and cultural backgrounds, the Chinese version of the BIDQ was submitted to a committee comprised of 4 native Chinese-speaking bilingual experts in related fields.

Experts evaluated the cultural aspects and language of the scale and made some recommendations for changes. On the third item (BIDQ3), the “defects” were supplemented, and on BIDQ4, the occupation and social sections were adjusted to make them easier to read.

### 2.6 Pilot test

In October 2017, 10 patients were recruited to complete the preliminary version of the scale as a means of understanding their experiences.

### 2.7 Ethical review

The study was approved by the ethics committee of Affiliated Hospital 2 of Nantong University (2017-016). All patients who met the criteria were invited to participate. They were informed of the purpose and importance of this study, the special emphasis on confidentiality, and that their involvement was completely voluntary and they could withdraw from the study at any time. All questionnaires were kept confidential.

### 2.8 Data collection

We standardized the training process to reduce bias and to ensure that researchers collected the data uniformly. After receiving information regarding the study, both in written and verbal form, all the participants gave their informed consent. Patients received face-to-face information from the researchers as well as guidance regarding how to complete the questionnaires. The researchers asked the participants to respond to each item on the scale in a sequence before recording their response objectively. To evaluate the test-retest reliability of the BIDQ, 35 patients completed the scale a second time. The second administration of the scale was conducted 4 weeks after the first.

### 2.9 Statistical analyses

We used SPSS version 24.0 and Amos version 22.0 for statistical analyses, and *P* < .05 was significant. We summarized the demographic characteristics of the patients using descriptive statistics and calculated means and standard deviations for descriptive statistical analysis of the items. Further screening of items was done through item-total score correlation analysis and the critical ratio, and we determined the content validity of the questionnaire through expert evaluation and exploratory factor points. A factor analysis was conducted to evaluate the structural validity of the questionnaire, and we used Cronbach's α and test-retest reliability coefficients to evaluate the scale's reliability.

## 3 Results

A total of 169 patients with SLE were invited to participate in this study, and 161 (95.27%) were eventually included in the present research.

### 3.1 Participant characteristics

Table 1 shows baseline participant characteristics in this analysis. All the participants were women and had a mean age of 37.35 years (SD = 11.41). The disease duration of participants was 7.41 years (SD *=* 6.39). Slightly less than half (43.5%) lived in the city. Most (81.4%) were married. Around half (49.7%) of the patients had less than 9 years of education, 54.7% were currently employed, 73.3% had medical insurance, and 29.8% had yearly income less than RMB 15000.

**Table 1:** Characteristics of patients with SLE.

Characteristics	N/Mean	%/SD
Age	37.35	11.41
Disease duration (years)	7.41	6.39
Location		
City	70	43.5%
Town	91	56.5%
Marital status		
Married	131	81.4%
Unmarried	30	18.6%
Education		
≤9 years	80	49.7%
>9 years	81	50.3%
Work status		
Employed	88	54.7%
Unemployed	73	45.3%
Personal health insurance		
Yes	118	77.3%
No	43	26.7%
Yearly Income (RMB)		
<15000	48	29.8%
15000–33000	70	43.5%
>33000	43	26.7%
Smoking Use		
Yes	2	1.2%
No	159	98.8%
Alcohol Use		
Yes	5	3.1%
No	156	96.9%

### 3.2 Item analysis and content validity

Item analysis includes the computations of item-total correlation and the critical ratio. The Chinese version of the BIDQ is interpreted according to the total score, high score (top 27%), and low score (bottom 27%) response groups. The score of the top 27% was 39.04 (SD *=* 5.88), and the mean score of the bottom 27% was 12.85 (SD *=* 4.32). An independent sample *t*-test determined that the difference between the scores of each item in the high group and the low group was statistically significant (*P* < .01). Importantly, the 95% CI did not contain 0, indicating that the items had a high degree of discrimination. The correlation between the score of each item and the total score was *r* *=* .41–.60. The correlation coefficients between the items and the total score were all *r* > .40, *P* < .01, reaching significance, which suggested that the Chinese BIDQ items had good discrimination abilities and that all items should be retained.

Furthermore, professional experts were invited to independently evaluate the content validity of the Chinese version of the BIDQ. This evaluation yielded a CVI of .92, which was > .80 on the scale, indicated that the scale had good content validity.

### 3.3 Structural validity

#### 3.3.1 Kaiser-Meyer-Olkin (KMO) test, Bartlett's spherical test and the EFA

The Kaiser-Meyer-Olkin test (KMO) value and Bartlett's test results were evaluated before the EFA to ascertain whether the factor analysis was appropriate. Then, we conducted the EFA using principal component analysis with varimax rotation. Factor extraction and retention criteria were:

1. factor loading >.40,
2. eigenvalue >1.00, and
3. deleted items having a cross-loading >.10.^[16]^

The results showed that the KMO value of this study was .75, which was greater than .50, indicating that the partial correlation between the variables was weak. Bartlett's spherical test was χ^2^ = 159.70 (*P* < .001), suggesting that the factor analysis could explain most of the information content of the scale's items. The principal component analysis method and the maximum variance rotation method were used to extract common factors without limiting the number of factors. According to the feature value >1, 2 common factors were extracted. The attribution of each entry's factor is consistent with the original scale. Factor 1 included Items 3, 4, 5, 6, and 7 and is named “effects.” Factor 2 included Items 1 and 2 and is named “attention and intervention.” The cumulative variance contribution rate of the 2 common factors was 67.83%. Factor loadings for all items were >.70, which indicated good structural validity (Table 2).

**Table 2:** The loading value, commonality, eigenvalue, contribution rate and cumulative contribution rate of each item in the Chinese version of BIDQ (n = 54).

	Factor 1	Factor 2
BIDQ1	.10	.78
BIDQ2	.02	.85
BIDQ3	.75	.18
BIDQ4	.81	.17
BIDQ5	.82	.00
BIDQ6	.86	.03
BIDQ7	.84	-.04
Eigenvalues	3.42	1.33
Variance contribution rate (%)	48.80	19.03
Cumulative variance contribution rate (%)	48.80	67.83

#### 3.3.2 CFA

**Figure 1:**
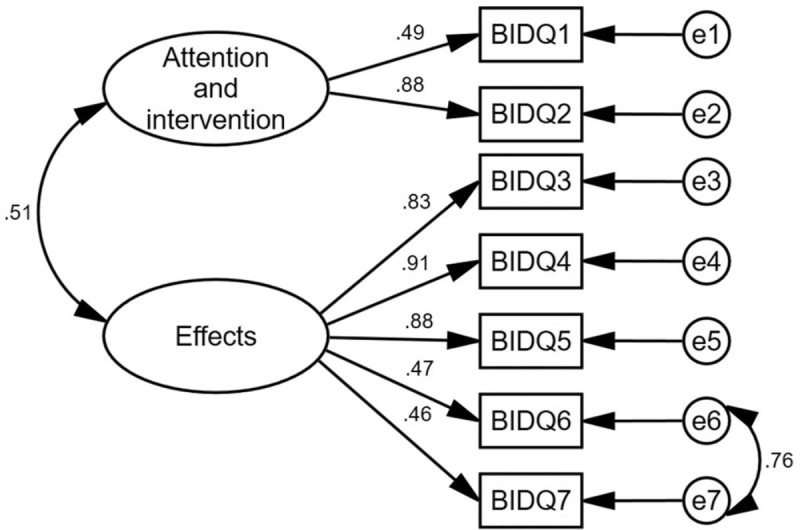
The modified model of the BIDQ (N = 117). Based on the adaption from the EFA, the two-factor structure was confirmed with another independent sample through CFA. The factor loading of all items ranged from 0.46 to 0.91 in CFA and met the acceptable level. BIDQ = Body Image Disturbance Questionnaire, EFA = exploratory factor analysis, CFA = confirmatory factor analysis.

We evaluated the structural validity of the scale by conducting CFA. In the initial model, the relevant indicators did not reach model fit guidelines, which led to modifications in the model. The results of the revised model and related indicators appear in Table 3 and Figure 1. Some of the main indicators of the revised model are the χ^2^/df = 1.79, root mean square error of approximation = .086, goodness-of-fit index = .95, normed fit index = .95, comparative fit index = .98, Tucker-Lewis index = .96, and incremental fit index = .98. All these indicators have reached acceptable model fit guidelines. The model fit was improved, indicating that the model had good structural validity. The factor loading of all items ranged from 0.46 to 0.91 in CFA and reached an acceptable level (Fig. 1).

**Table 3:** The results of the confirmatory factor analysis (N = 117).

Fit index	Criteria	Initial model	Modified model
χ^2^/df	<3	8.46	1.79
RMSEA	<.09	.27	.09
GFI	>.9	.80	.95
NFI	>.9	.74	.95
CFI	>.9	.75	.98
TLI	>.9	.60	.96
IFI	>.9	.76	.98

### 3.4 Reliability

The Cronbach's α for the overall BIDQ was .82 (Cronbach's α >.80), with the Cronbach's α for each item ranging from .76 to .83 (Cronbach's α > .70). The test-retest reliability was .82, with the Cronbach's α for each item ranging from .76 to .84.

## 4 Discussion

Facial erythema, joint pain, deformity, hair loss, tooth loss, scars, hairiness, and weight gain often accompany the presentation and disease course of SLE. These factors could contribute to physical image disorders in patients with SLE. The symptoms of this disease are very visible; therefore, the patient's subjective feelings about their body image are negatively affected. These changes could contribute to concerns and problems in patients’ daily lives. Owing to changes in their appearance, patients with SLE sometimes have unpleasant experiences in interpersonal communication, which could lead them to use avoidance behaviors and reduce their social interactions. These experiences might place pressure on patients with SLE, reduce their self-esteem, produce negative emotions, increase the occurrence of anxiety and depression, and reduce their quality of life.

Currently, research on body image has been primarily focused on patients with breast cancer.^[17]^ In 1 study of patients with cancer, a well-developed and complete plan for a body image intervention for patients with cancer was developed and achieved promising results.^[18]^ Thus, interventions that target the body image and health outcomes of patients with SLE could be effective.^[19,20]^ However, at present, the field's understanding of the perceived body image of patients with SLE in China is in its infancy and is lacking the necessary methods of assessment, making it impossible to evaluate the specific degree of the BID clearly.

This study shows that the BIDQ is a valid and reliable measure. In this study, the scale translation and reverse translation phases were discussed in groups. To further adapt to the local culture and improve the applicability of the scale, we conducted consultations with experts before the survey and improved the content based on the results of the survey and expert suggestions. Further, we tested the discriminability of the scale by calculating the item-by-item correlation and critical ratio. The results showed that the discriminative power of the scale was good. Our analysis showed that the scale has good reliability and validity.

BIDQ currently has a British version,^[21]^ an Australian version,^[22]^ and a Malaysian version^[23]^ that also have high-quality clinical research significance. However, body image is affected by many factors. Different countries and regions have different aesthetic standards and aesthetic cultures, so their residents will have different experiences, and their BIDQ scores will also be different. In a study by Brockhoff and colleagues^[24]^ that compared samples from China, Malaysia, Tonga, Fiji, and Australia, although Japanese adolescents have the lowest body mass index, their dissatisfaction with their bodies is the highest, and the media's influence on their body image is also the highest. Subsequent path analysis showed that, for Japanese teenagers, the cultural identity of modern Japanese values is associated with increased dissatisfaction with the body, and this association is regulated by the degree of media influence. The results highlight the importance of cultural influences and individual differences in cultural values in shaping one's body image. Of course, this hypothesis may require further research data for confirmation.

In patients with SLE, the SLE symptom checklist was included to assess disease-specific quality of life; this is mainly a symptom scale.^[25]^ It contains the presence and burden of 38 symptoms, but it simply lists related symptoms and does not include other manifestations related to body image. There are two questionnaires, the Lupus Quality of Life (LupusQoL)^[26]^ and the systemic lupus erythematosus-specific quality-of-life questionnaire (SLEQoL).^[27]^ Although the content involves body image, the tool is not sufficiently comprehensive. Studies have shown that among women,^[22]^ the BIDQ score most strongly assesses the degree of body image disturbance, dysfunction, and social environment interference. SLE patients are mostly young women, so they have a deeper experience.

It is reported that the BID of the SLE/Ankylosing spondylitis/Sjögren's syndrome group^[4,12,28]^ was significantly higher than that of the control group. At the same time, individuals with RA had a worse body image than individuals without this condition.^[29]^ Body-image–related quality of life may mediate the effects of pain on depressive symptoms among patients with SLE,^[30]^ serving as a reminder that body image disorders in patients with rheumatism need special attention. With regard to time, it takes about 5 minutes to fill out the scale, a short duration, which makes it suitable for clinical use. When researchers assess the degree of a body image disorder, BIDQ can be used for rapid assessment, and at the same time, the judgment of the intervention effect can also be quickly determined. Therefore, doctors may find BIDQ useful as a simple and reliable method of identifying important body image problems, as well as a simple way to track treatment across its courses.

The generalization of the study's results may be limited for several reasons. First, it is important to mention that the sample could have been more widely represented. However, the prevalence of SLE is relatively low, which limited the available data that could be collected. Thus, future research should expand the sample size. Second, since body image disorders are a highly subjective target of analysis, the next step is to add objective measurements.

In conclusion, this paper demonstrates that the Chinese version of the BIDQ had good reliability and validity in this sample of patients with SLE. The scale can be promoted for use among Chinese patients with SLE and can provide a foundation for future research in the development of body image interventions.

## Author contributions

**Conceptualization:** Haoyang Chen, Xin Chen, Biyu Shen.

**Data curation:** Haoyang Chen, Xin Chen, Pingyu Yang, Biyu Shen.

**Formal analysis:** Hongyan Yan, Biyu Shen.

**Funding acquisition:** Biyu Shen.

**Investigation:** Haoyang Chen, Xin Chen, Hongyan Yan, Jielin Ben, Xiaoyan Yao, Pingyu Yang, Minhua Zhang, Tiantian Jin, Biyu Shen.

**Methodology:** Haoyang Chen, Xin Chen, Hongyan Yan, Xiaoyan Yao, Minhua Zhang, Tiantian Jin, Biyu Shen.

**Project administration:** Jielin Ben, Biyu Shen.

**Resources:** Hongyan Yan, Jielin Ben, Minhua Zhang, Biyu Shen.

**Supervision:** Hongyan Yan, Jielin Ben, Biyu Shen.

**Validation:** Xiaoyan Yao, Biyu Shen.

**Visualization:** Xiaoyan Yao, Biyu Shen.

**Writing – original draft:** Haoyang Chen, Xin Chen, Biyu Shen.

**Writing – review & editing:** Haoyang Chen, Xin Chen, Biyu Shen.
